# Biodegradable MXene‐Bamboo Cellulose Paper Electrodes for Green Wearable Sensing and Exoskeleton Control

**DOI:** 10.1002/advs.202509554

**Published:** 2025-09-11

**Authors:** Tung‐Li Hung, Chien‐Yu Huang, Chun‐Ho Lin, Yu‐Chen Wei, Yung‐Jung Hsu, Jr‐Hau He, Yun‐Ting Kuo, An‐Yu Huang, You‐Yin Chen, You‐Rong Lin, Clemens M. Franz, Chia‐Hao Kuo, Pulikkutty Subramaniyan, Shan‐Chu Yu, Xinwei Guan, Tzu‐En Lin

**Affiliations:** ^1^ Institute of Biomedical Engineering National Yang Ming Chiao Tung University Hsinchu 300093 Taiwan; ^2^ School of Materials Science and Engineering University of New South Wales Sydney NSW 2052 Australia; ^3^ Department of Materials Science and Engineering National Yang Ming Chiao Tung University Hsinchu 300093 Taiwan; ^4^ Center for Emergent Functional Matter Science National Yang Ming Chiao Tung University Hsinchu 300093 Taiwan; ^5^ Institute of Integrated Research Institute of Science Tokyo Kanagawa 226–8503 Japan; ^6^ Department of Materials Science and Engineering City University of Hong Kong Kowloon Hong Kong SAR 53025 China; ^7^ Department of Biomedical Engineering National Yang Ming Chiao Tung University Taipei 112304 Taiwan; ^8^ WPI Nano Life Science Institute Kanazawa University Kanazawa 920–1192 Japan; ^9^ Institute of Applied Mechanics National Taiwan University Taipei 10067 Taiwan; ^10^ School of Engineering Macquarie University Sydney NSW 2109 Australia

**Keywords:** bamboo cellulose nanofiber, exoskeleton, green electronics, MXene, wearable devices

## Abstract

The global rise in electronic waste highlights the urgent need for green electronics that minimize environmental impact through sustainable material selection and fabrication methods. In this work, multifunctional, biodegradable paper electrodes, designated as MXN_x_/B‐CP, are prepared via a simple vacuum‐assisted assembly of homogenized MXene (Ti_3_C_2_T_x_) nanosheets within bamboo‐derived cellulose nanofiber (CNF). These freestanding paper electrodes offer tunable electrical conductivity, mechanical flexibility, and low‐cost, scalable production. To enhance their stability, the electrodes are encapsulated in a breathable, porous Ecoflex layer, which imparts waterproofing while maintaining gas permeability. Strong hydrogen bonding at the MXene‐CNF interface facilitates continuous electron transport and structural integrity, yielding a nonlinear piezoresistive response with a gauge factor increasing from 3.7 to 11.42 at small strain range, alongside a strain‐adaptive Young's modulus ranging from 0.064 to 1.768 MPa. Benefiting from this synergistic design, the electrodes support a wide range of sensing applications, including bending strain detection, surface electromyography, and human‐machine interfaces for exoskeleton control while exhibiting excellent stability, low noise, and long‐term durability under repeated deformation. This innovation not only expands the potential of paper‐based electronics but also offers a scalable pathway toward sustainable, high‐performance solutions for next‐generation wearable and assistive technologies.

## Graphical abstract

A biodegradable, soft, and conductive MXene‐cellulose nanofiber paper electrode integrates Ti_3_C_2_T_x_ nanosheets into bamboo‐derived scaffolds, with a porous Ecoflex coating that imparts waterproofing and breathability. The freestanding dry electrode enables high‐fidelity EMG sensing, strain and pressure detection, and wireless control of a knee exoskeleton. Upon disposal, the material degrades oxidatively, offering a sustainable route toward scalable, eco‐friendly human‐machine interfaces.

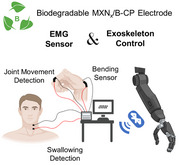

## 1 Introduction

The rapid development of wearable electronics is reshaping prosthesis control, rehabilitation monitoring, and human‐machine interfaces^[^ ^1^, ^2^, ^3^, ^4^ ^]^ through skin‐conformal sensors that transduce biomechanical and bio‐electrical cues into digital commands. Among these, surface electromyography (EMG) stands out as a key noninvasive tool for monitoring muscle activity and is widely used in clinical diagnostics.^[^ ^5^ ^]^ Despite considerable progress in enhancing comfort, safety, and biocompatibility of wearable electronics,^[^ ^6^, ^7^ ^]^ today's single‐use Ag/AgCl EMG electrodes that rely on dehydrating hydrogels and nondegradable plastic films, have not only posed challenges in long‐term wearability and skin compatibility,^[^ ^8^, ^9^ ^]^ but also contributed significantly to electronic waste (e‐waste) accumulation. In 2022 alone, an estimated 62 million tonnes of e‐waste were generated worldwide,^[^ ^10^ ^]^ underscoring the urgent need for green electronic systems.

Central to the evolution of green electronics is the strategic integration of biodegradable, environmentally friendly materials with cost‐effective fabrication approaches.^[^ ^11^ ^]^ Among various candidates, cellulose has garnered widespread attention as a renewable, naturally abundant biopolymer offering impressive mechanical strength and biodegradability.^[^ ^12^ ^]^ Advances in nanotechnology now enable the effective isolation of cellulose nanofibers/nanocrystals (CNFs/CNCs) from fast‐growing plants such as bamboo,^[^ ^13^ ^]^ yielding nanoscale building blocks rich in surface hydroxyl groups, with high interface activity and the ability to form self‐supporting films of outstanding mechanical strength.^[^ ^14^, ^15^ ^]^ In parallel, embedding functional nanomaterials within CNF scaffolds has proven effective for marrying environmental compatibility with electronic functionality. Our group pioneered a room‐temperature vacuum filtration strategy to embed 2D boron nitride (h‐BN) and borophene into 1D CNFs, forming mechanically robust and flexible nanopapers, demonstrating the versatility of cellulose as a green and structurally adaptive platform for nanomaterial integration.^[^ ^16^, ^17^ ^]^ However, the practical application of these materials in active sensing remains constrained, as h‐BN is electrically insulating and borophene is prone to rapid oxidation.

Recent advances in electrically conductive nanomaterials such as carbon nanotubes (CNTs),^[^ ^18^ ^]^ silver nanowires,^[^ ^19^ ^]^ and graphene,^[^ ^20^ ^]^ have significantly expanded the operational ranges and mechanical compliance of sensor technologies beyond what is achievable with conventional bulk counterparts. In particular, 2D MXenes (e.g., Ti_3_C_2_T_x_) have attracted significant interest for their outstanding electrical conductivity, hydrophilicity, and biocompatibility,^[^ ^21^, ^22^ ^]^ enabling them to be employed in pressure sensors,^[^ ^23^ ^]^ triboelectric nanogenerators,^[^ ^24^ ^]^ electromagnetic interference shielding,^[^ ^25^ ^]^ and hybrid sensors.^[^ ^26^, ^27^, ^28^, ^29^, ^30^ ^]^ Several recent studies have attempted to combine MXene with biodegradable matrices, such as CNF foam,^[^ ^31^ ^]^ aerogels,^[^ ^32^ ^]^ and sponges,^[^ ^33^ ^]^ to develop soft, flexible sensors for mechanical or strain detection. Nonetheless, no study has delivered a freestanding, fully degradable MXene/CNF electrode that maintains low skin‐electrode impedance under perspiration and repetitive flexion, functions reliably underwater, and directly actuates a powered exoskeleton in real time. Existing MXene sensors are typically characterised only for mechanical deformation; their EMG fidelity and system‐level human‐machine integration remain unexplored.

In this study, we present a sustainable, skin‐compatible, and multifunctional sensing platform by integrating Ti_3_C_2_T_x_ nanosheets into bamboo‐derived CNF paper (MXN_x_/B‐CP, where x denotes MXene loading) through a facile vacuum filtration process. Designed with an eco‐conscious approach, the electrode features a porous, biodegradable, and compostable Ecoflex coating that confers waterproofing while retaining gas permeability and biodegradability. More importantly, the intimate integration of MXene into CNFs enhances durability and enables tunable conductivity, supporting reliable electrophysiological and mechanical sensing across diverse conditions. The MXN_x_/B‐CP electrodes were applied in strain sensors, underwater motion trackers, and EMG‐driven interfaces for a knee exoskeleton, reducing rectus‐femoris activation during assisted gait. Our primary focus is on EMG sensing technologies to support individuals in need of physical assistance. This work advances the frontier of truly green wearable robotics by uniting degradability, mechanical adaptability, and system‐level human‐machine interface integration.

## 2 Results and Discussion

### 2.1 Synthesis and Structural Characterizations

**Figure 1:**
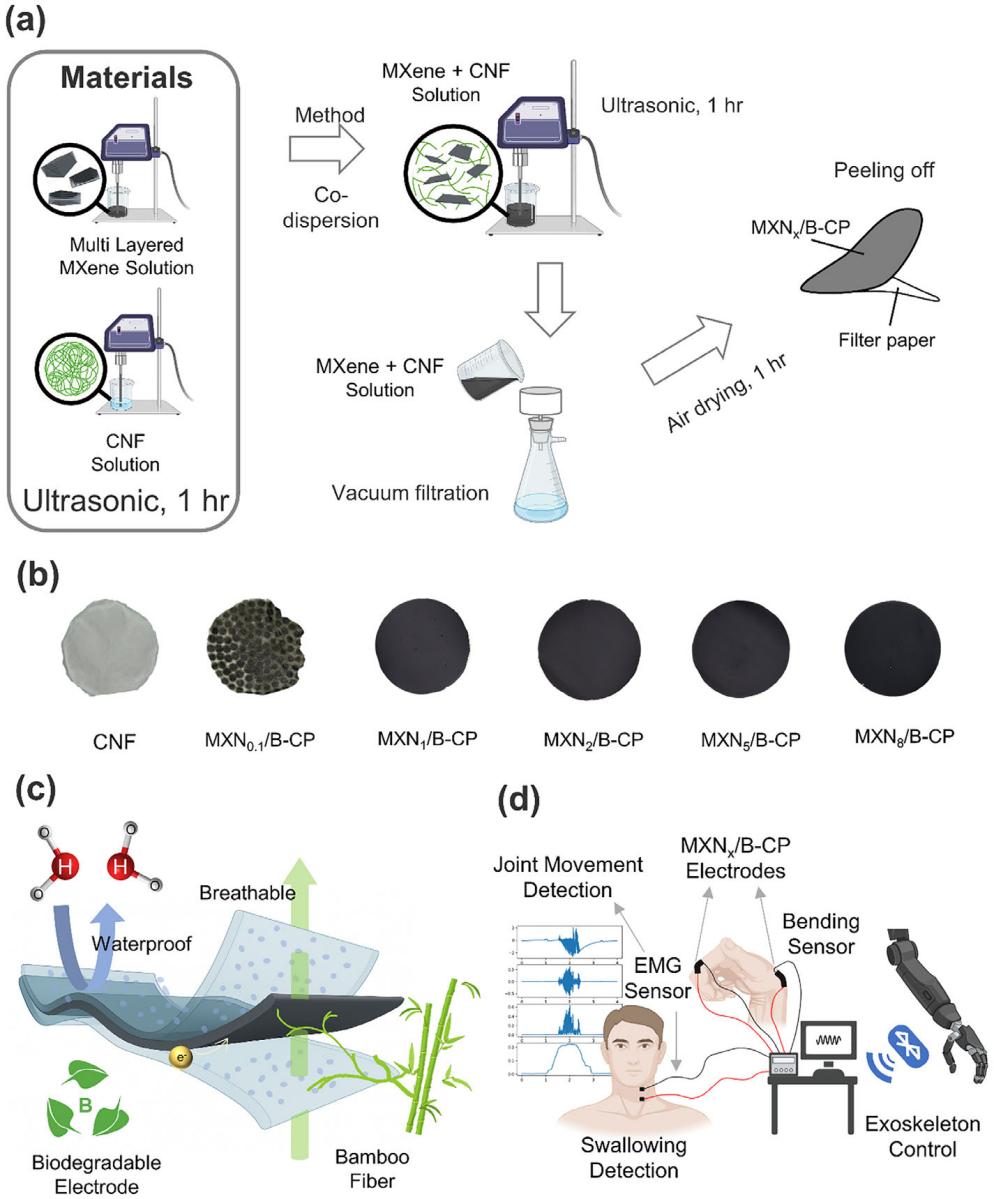
a) Schematic illustration of the synthesis process of MXN_x_/B‐CP. b) Digital photographs showing the visual appearance of MXN_x_/B‐CP with different MXene loadings. c) Schematic illustration of the waterproof and breathable MXN_x_/B‐CP structure enabled by a porous Ecoflex coating. d) Demonstration of MXN_x_/B‐CP applications as EMG sensors for swallowing detection, joint movement sensing, and human‐machine interfaces for exoskeleton control.

The detailed synthesis procedures of MXN_x_/B‐CP are illustrated in **Figure** **1**a. In brief, recycled wastepaper containing bamboo fiber was processed into pulp, followed by TEMPO (2,2,6,6‐Tetramethylpiperidin‐1‐yl)oxyl‐mediated oxidation to introduce carboxyl (‐COOH) groups onto the CNFs. The oxidized CNFs were further disintegrated by a microfluidizer to obtain a fine CNF suspension composed of 1D fibrils. In parallel, multilayered MXene powders were delaminated into single‐stacking nanosheets following the protocol established in our previous work.^[^ ^34^ ^]^ In the co‐dispersion step, pre‐exfoliated aqueous MXene was thoroughly mixed with the CNF suspensions under ice‐bath conditions using a probe sonicator, ensuring homogeneous dispersion. Afterward, the mixed solution was vacuum‐filtered and air‐dried at room temperature for 1 h to fully remove residual moisture. Finally, the as‐prepared MXN_x_/B‐CP were carefully peeled off from the filter membrane for subsequent characterization and device fabrication.

In order to modulate the mechanical compliance and electrical conductivity of MXN_x_/B‐CP, varied amounts of MXene powder (i.e., 0, 0.1, 1, 2, 5, and 8 mg; see **Table** **1**) were co‐dispersed with a fixed bamboo‐CNF suspension during paper fabrication. As shown in Figure 1b, the MXN_x_/B‐CPs demonstrate a distinct color evolution from off‐white (pure CNF) to jet‐black (MXN_8_/B‐CPs) as the percolating MXene network develops, visually confirming successful, composition‐dependent incorporation. To enhance environmental adaptability, the conductive MXN_x_/B‐CP was encapsulated with a porous Ecoflex thin film (Figure 1c), in which the interconnected pores create a tortuous path that blocks liquid water while permitting vapour diffusion, imparting the electrode with simultaneous waterproofing and gas permeability. Leveraging its unique architecture and functionalities (Figure 1d), the resulting MXN_x_/B‐CP electrode is suitable for diverse applications, including EMG sensors (swallowing, joint movement detection), and human‐machine interfaces for exoskeleton control.

**Table 1:** Material composition of different MXN_x_/B‐CP.

Procedures	MXene	CNF	Abbreviation
Co‐dispersion	0 mg mL^−1^	0.55 mg mL^−1^	CNF
	0.1 mg mL^−1^	0.55 mg mL^−1^	MXN_0.1_/B‐CP
	1 mg mL^−1^	0.55 mg mL^−1^	MXN_1_/B‐CP
	2 mg mL^−1^	0.55 mg mL^−1^	MXN_2_/B‐CP
	5 mg mL^−1^	0.55 mg mL^−1^	MXN_5_/B‐CP
	8 mg mL^−1^	0.55 mg mL^−1^	MXN_8_/B‐CP

**Figure 2:**
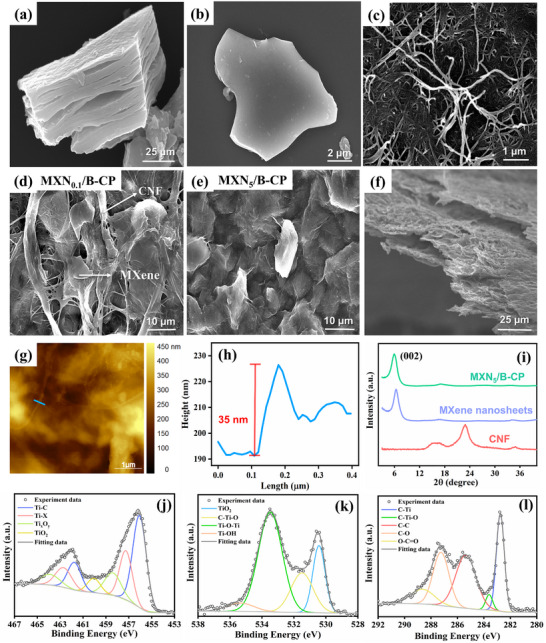
SEM images of a) multilayered MXene nanosheets, b) single or few‐layer MXene nanosheets, c) bamboo‐derived CNF nanofibers, and d,e) MXN_x_/B‐CP ((d) MXN_0.1_/B‐CP, (e) MXN_5_/B‐CP) with different concentrations of MXene. f) Cross‐section SEM image of the MXN_5_/B‐CP. g) AFM image of MXN_5_/B‐CP. h) Structural height profile along the line blue in g). i) XRD pattern of MXN_5_/B‐CP, CNF, and MXene nanosheets. High‐resolution XPS spectra of j) Ti 2p, k) O 1s, and l) C 1s in the MXN_5_/B‐CP composite.

As expected, the MXN_x_/B‐CP exhibits outstanding flexibility and mechanical robustness, which can be folded into various shapes, such as a paper boat (Figure S1a, Supporting Information) and Miura origami folding structures (Figure S1b, Supporting Information), demonstrating its excellent crimpability, tailorability, and foldability. The folded MXN_5_/B‐CP boat is capable of floating on water (Figure S1c, Supporting Information), indicating its low density and structural integrity. Scanning electron microscope (SEM) analysis characterized the morphology of the constituent materials. Multilayered bulk MXene exhibited stacked lamellar structures (**Figure** **2**a), while post‐sonication delaminated it into few‐layer 2D nanosheets (Figure 2b). The pure CNF paper exhibited a network of 1D nanofiber structure (Figure 2c); upon the incorporation of MXene nanosheets, the resulting MXN_x_/B‐CP films displayed well‐integrated architectures with visible 2D/1D hybrid structures, as shown in Figure 2d,e (MXN_0.1_/B‐CP, and MXN_5_/B‐CP) and Figure S1d–f (Supporting Information). In this paper, the architecture, 2D MXene nanosheets aligns and stacks along the planar axis of the CNF network, facilitating large contact areas and efficient electron transport across adjacent nanosheets. This hierarchical integration of MXene and CNF underpins the film's high conductivity, mechanical robustness, and foldability.

Following a preliminary screening of the six compositions (**as discussed in** **Section** **2.2**), we selected MXN_5_/B‐CP, which exhibited the best balance of conductivity and mechanical properties, for detailed structural and functional characterization. The elemental mapping (Figure S1g–i, Supporting Information) confirms the homogeneous distribution of oxygen, titanium, and carbon in the MXN_5_/B‐CP, supporting the uniform integration of MXene and CNF. The cross‐sectional SEM image of the MXN_5_/B‐CP (Figure 2f) further reveals the layered yet cohesive architecture of the composite. AFM was employed to investigate the surface morphology and nanoscale roughness of the MXN_5_/B‐CP (Figure 2g), revealing a heterogeneous but continuous surface landscape composed of entangled nanofibrils and embedded MXene sheets, consistent with the SEM observations in Figure 2d. A representative height profile taken along the marked scan line shows a distinct peak of 35 nm (Figure 2h), which is attributed to the characteristic thickness of stacked MXene nanosheets. These characterizations confirm that the MXene sheets are uniformly embedded within the CNF matrix, creating a continuous conductive network. Such an architecture is critical for achieving high electrical conductivity and mechanical flexibility in the final device.

The crystallinity and phase composition of the individual components and the composite were confirmed through XRD analysis (Figure 2i). The observed 2θ peaks at 16° and 22°, corresponding to the crystalline structure of CNF, were observed in the pure CNF sample.^[^ ^35^, ^36^ ^]^ Meanwhile, a prominent peak at 6° aligns with the (002) plane of hexagonal Ti_3_C_2_T_x_. In the composite MXN_5_/B‐CP, the strong MXene (002) reflection was dominant, while the typical CNF peaks were not distinctly observed, likely due to the high MXene content, which may lead to the lattice plane disruption of CNF. Nevertheless, the presence of the (002) MXene peak in the composite, along with the uniform morphology observed in SEM, confirms the successful integration of MXene into the CNF matrix.^[^ ^37^, ^38^ ^]^ XPS analysis provides critical insights into the chemical interactions and bonding configuration within the MXN_5_/B‐CP composite. High‐resolution XPS spectra for Ti 2p, O 1s, and C 1s are presented in Figure 2j–l. The Ti 2p spectrum in Figure 2j can be deconvoluted into three sets of doublets (Ti 2p_3/2_ and Ti 2p_1/2_). The Ti 2p_3/2_ peaks are located at 456.0, 457.2, and 458.4 eV, which correspond to Ti‐C, Ti‐X from Ti(II) or titanium oxycarbides, and Ti ions in a reduced charge state (Ti_x_O_y_), respectively.^[^ ^39^, ^40^ ^]^ Additionally, the peak at 460.0 eV is attributed to the TiO_2_ component.^[^ ^41^ ^]^ The O 1s spectrum (Figure 2k) exhibits peaks at 530.4, 531.5, 533.5, and 535.3 eV, which can be assigned to TiO_2_, C‐Ti‐O, Ti‐O‐Ti, and Ti‐OH bonds, respectively.^[^ ^42^ ^]^ In the C 1s spectrum (Figure 2l), multiple deconvoluted peaks appear at 282.7, 283.6, 285.3, 287.2, and 288.6 eV, which correspond to C‐Ti and C‐Ti‐O bonds from MXene, along with C‐C, C‐O, and O‐C = O functional groups from CNF.^[^ ^43^, ^44^, ^45^ ^]^ The presence of abundant hydroxyl (C–OH) groups on MXene and CNFs promotes strong hydrogen bonding interactions, facilitating robust bonding between the two components. These interactions contribute to the mechanical cohesion and structural integrity of the MXN_5_/B‐CP composite, while the preserved chemical state of MXene ensures that its intrinsic properties are retained within the composite matrix.

### 2.2 Electrical and Mechanical Properties

**Figure 3:**
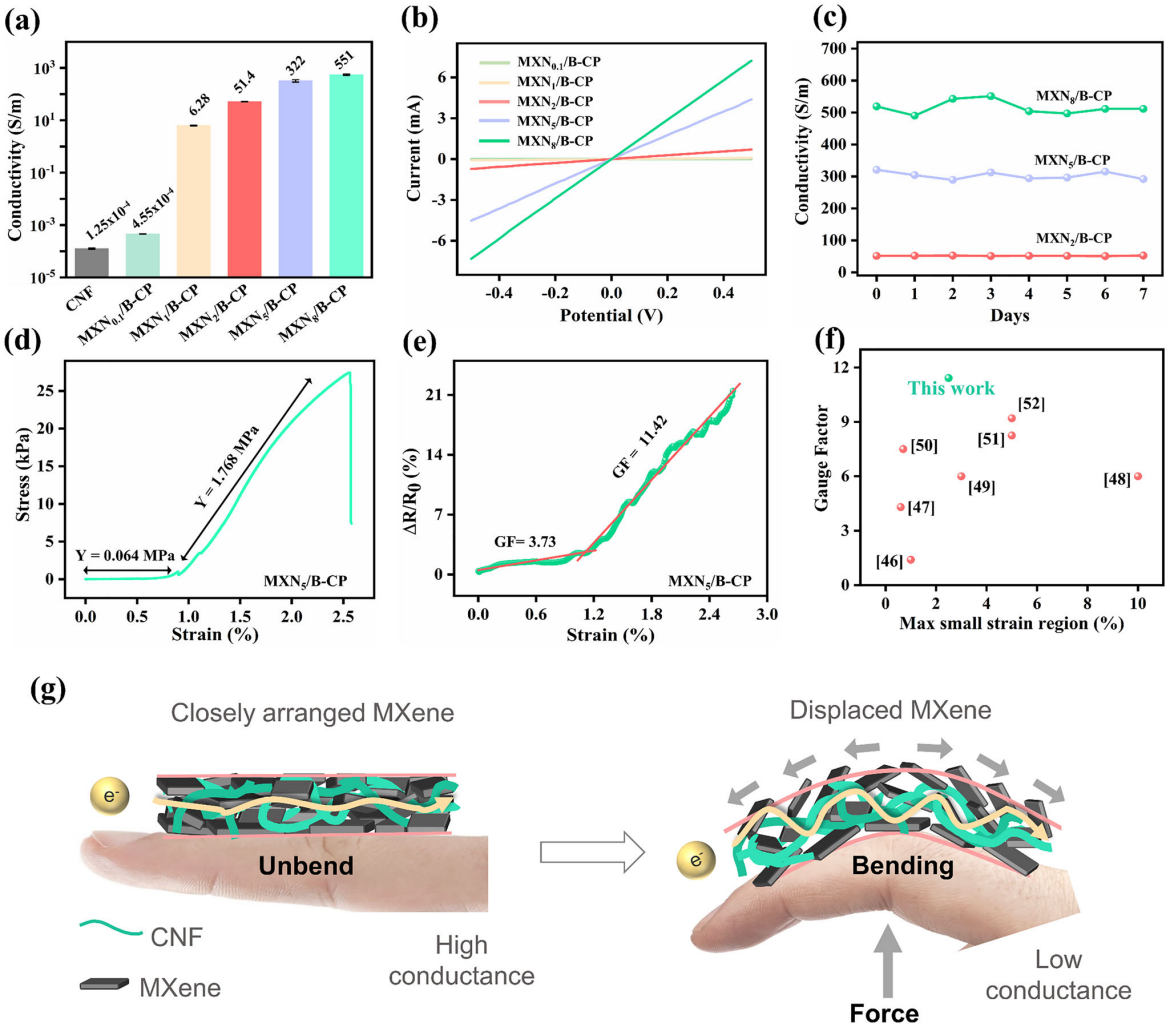
a) Electrical conductivity of CNF and MXN_x_/B‐CP papers with varying MXene content. b) LSV curves of different MXN_x_/B‐CP papers. c) Time‐dependent conductivity of MXN_2_/B‐CP, MXN_5_/B‐CP, and MXN_8_/B‐CP exposed to ambient air for one week. d) Tensile stress–strain curve of MXN_5_/B‐CP paper. e) Relative resistance changes of MXN_5_/B‐CP paper under applied strain. f) Gauge factor comparison of our MXN_5_/B‐CP strain sensors with reported literature in the small‐strain region. g) Schematic illustration of structural changes within MXN_x_/B‐CP during bending deformation.

Following confirmation of the composite's successful structural integration, we next investigated the electrical and mechanical properties of the MXN_x_/B‐CP, including its conductivity, strain‐dependent response, and mechanical stability. These properties are essential to the device's sensing accuracy and robustness under everyday deformation conditions. As the MXene content increased, the paper color gradually changed from white to black (Figure 1b), correlating with a marked enhancement in the electrical conductivities (**Figure** **3**a) from an insulating state for pure CNF and MXN_0.1_/B‐CP to ≈50, 300, and 550 S m^−1^ for MXN_2_/B‐CP, MXN_5_/B‐CP, and MXN_8_/B‐CP, respectively, attributing to the gradual construction of interconnected conductive MXene networks. Corresponding linear I‐V plots (Figure 3b; Figure S2, Supporting Information) confirm the establishment of ohmic contacts for all MXene‐containing samples, indicating the successful construction of an interconnected conductive network. Moreover, the fabricated MXN_x_/B‐CPs demonstrate remarkable durability, maintaining stable conductivities over seven days under ambient conditions (Figure 3c). Among the tested samples, the MXN_5_/B‐CP sensor paper exhibited moderate conductivity and high stability, maintaining a consistent conductivity ranging from 320 to 290 S m^−1^. It retained 92% of its performance over seven days under open‐air conditions at room temperature, and was therefore selected for subsequent mechanical characterization.

Tensile testing was performed to evaluate the tensile strain‐dependent mechanical and piezoresistive behavior of the MXN_5_/B‐CP composite (Figure 3d). The strain–stress curve initially shows a linear elastic region, where stress (𝜎 = F/A, where F is the applied force (N) and A is the cross‐sectional area (m^2^)) increased proportionally with strain (𝜀 = ∆L/L_0_, where ∆L is the change in length and L_0_ is the original length), indicating a uniform deformation governed by Hooke's law:

(1) σ=Eε

where 𝜎 is the stress (Pa), E is Young's modulus (Pa), and 𝜀 is the strain (unitless). Afterward, the MXN_5_/B‐CP sensor exhibited a nonlinear elastic response, with Young's modulus increasing from 0.064 MPa in the low‐stress region to 1.768 MPa under higher stress. This pronounced strain‐induced stiffening behavior is particularly beneficial for wearable applications, where initial softness ensures conformability and subsequent stiffening provides mechanical integrity under dynamic loading. In contrast, the MXN_2_/B‐CP (Figure S3a, Supporting Information) sample displayed a higher Young's modulus of 0.408 MPa in the low‐stress region but exhibited a more moderate increase to 0.854 MPa at higher stress levels. This comparison highlights that MXN_5_/B‐CP offers a broader mechanical tunability, starting from a softer state and adapting to increased mechanical demands, whereas MXN_2_/B‐CP maintains relatively higher initial stiffness with less pronounced stiffening under strain.

The electromechanical response under tensile loading was evaluated by monitoring the relative resistance change (ΔR/R_0_) as a function of applied strain (Figure 3e). A progressive increase in resistance with strain was also observed, indicating a clear piezoresistive effect. This response could be attributed to the elongation‐induced microcrack formation or the change in conductive pathways within the composite. At lower strains, a gradual resistance change was noted due to reversible deformation and preservation of the conductive network, which is desirable for strain‐sensing applications with high sensitivity. Beyond a critical strain, the resistance exhibited a nonlinear escalation, suggesting the initiation of microcracks and partial disconnection of conductive networks within the MXene/CNF architecture, leading to a loss of electrical continuity. The gauge factor (GF) in two distinct strain regions is defined as follows:

(2) $$\mathrm{GF}\;=\frac{\Delta R/R_{0}}{\varepsilon }$$

In the lower strain region, the MXN_5_/B‐CP sensor exhibited a GF of 3.73, reflecting moderate sensitivity suitable for subtle motion detection. At higher strain levels, the GF increased significantly to 11.42, indicating a strong piezoresistive response resulting from dynamic deformations and rearrangements of conductive pathways. In contrast, the MXN_2_/B‐CP sample showed negligible piezoresistive sensitivity in the lower strain region, with the GF close to zero, suggesting that minor deformations did not effectively alter the conductive network. At higher strains, the GF of MXN_2_/B‐CP increased modestly to 2.33 (Figure S3b, Supporting Information), indicating a weak but positive strain response. This comparison highlights that MXN_5_/B‐CP delivers a much broader and more tunable sensitivity across different deformation regimes, making it more suitable for applications requiring reliable detection of both small and large mechanical strains, while MXN_2_/B‐CP demonstrates limited strain responsiveness, particularly under low deformation conditions. This dual‐mode behavior enhances the sensor's capability for both subtle and large‐scale motion detection. A performance comparison with previously reported literature in Figure 3f and Table S1 (Supporting Information) demonstrates that the MXN_5_/B‐CP sensor offers competitive or superior GF values across a small strain range, positioning it as a promising candidate for high‐precision, flexible sensing applications.^[^ ^46^, ^47^, ^48^, ^49^, ^50^, ^51^, ^52^ ^]^ As shown in Table S1 (Supporting Information), our MXN_5_/B‐CP sensor exhibits a strain‐active range that is 4 to 5 times broader and a sensitivity that is 2 to 3 times higher compared to other reported biodegradable materials.

The underlying sensing mechanism can be elucidated by a wide‐range microcracks model, as illustrated in Figure 3g. When the MXNx/B‐CP sensor is subjected to tensile stress (e.g., during bending), the sensor undergoes elongation similar to human skin. This mechanical deformation induces the generation and propagation of microcracks in the conductive MXene network. These microcracks disrupt the continuous conductive pathways, thereby increasing the resistance. Upon release, the cracks partially close, allowing the conductive network to recover, which results in a reversible resistance change. This behavior is in line with crack‐induced resistance modulation observed in other MXene‐ or microcrack‐based systems. Specifically, the deformation‐sensitive resistance arises from dynamic interfacial changes, where widened cracks act as barriers to electron transport, and the resistance increases (ΔR/R_0_) proportionally with strain due to reduced conductive pathways. When relaxed, the conductive islands reconnect, contributing to the sensor's stable and recoverable signal output. Such crack‐mediated piezoresistive response is well established in strain sensors designed for high sensitivity and flexibility, particularly in MXene‐based composites and microstructured elastomeric systems.^[^ ^53^, ^54^, ^55^, ^56^ ^]^

### 2.3 Assessment of Waterproofing, Flame Resistance, and Environmental Compatibility

**Figure 4:**
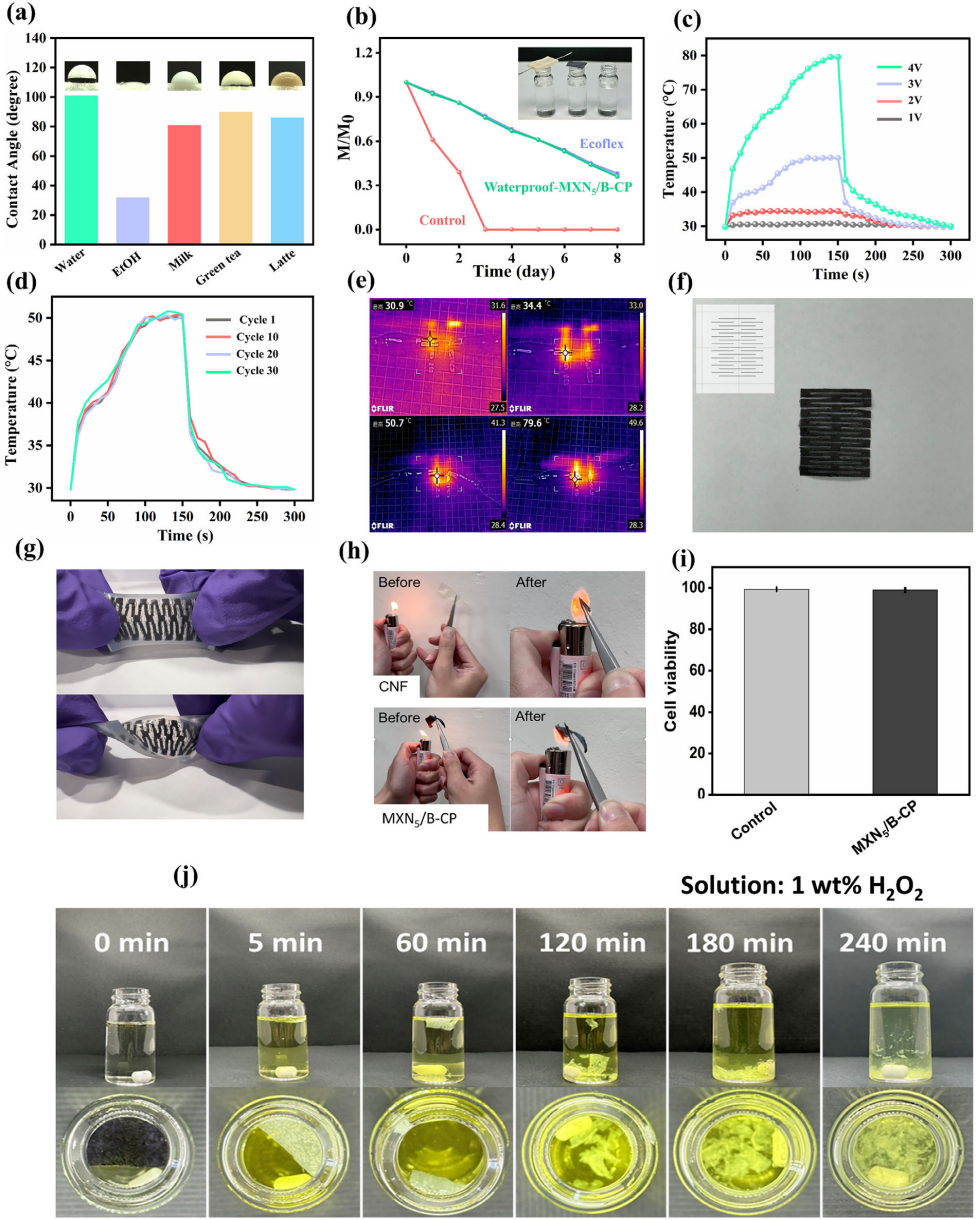
a) Contact angle measurements of various liquids (water, ethanol, milk, green tea, and coffee) on the surface of the waterproof MXN_5_/B‐CP. b) Moisture permeability experiment of sealed bottles using waterproofed MXN_5_/B‐CP, Ecoflex, and an unsealed control, with weight loss recorded at room temperature for 8 days. c) Joule heating performance of MXN_5_/B‐CP under applied voltages ranging from 1 to 4V. d) Stability of the Joule heating response of MXN_5_/B‐CP under 30 heating cycles at 3V. e) Infrared thermal images showing temperature rise of MXN_5_/B‐CP at 1, 2, 3, and 4 V. f) Customization of MXN_5_/B‐CP into a stretchable electrode via laser cutting. g) Demonstration of the waterproof electrode under mechanical stretching and twisting. h) Fire‐resistance experiment of pristine CNF paper and MXN_5_/B‐CP under direct flame. i) Cytotoxicity experiment of MXN_5_/B‐CP was conducted by co‐incubating with cells for 24 h. j) Degradation of MXN_5_/B‐CP in 1 wt.% of H_2_O_2_.

**Figure 5:**
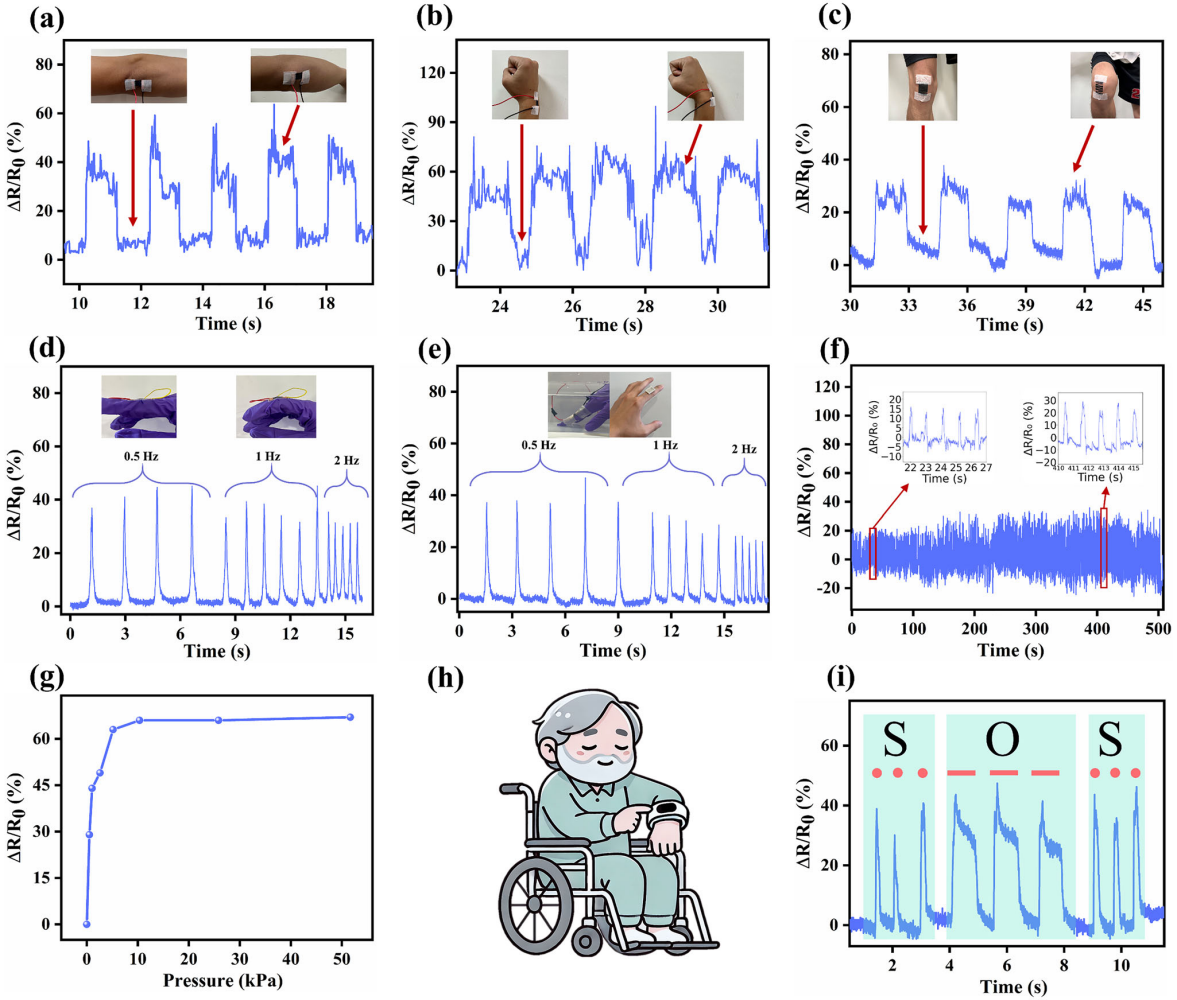
MXN_5_/B‐CP based strain sensor for human motion detection under various conditions. Real‐time detection of joint bending at a) elbow, b) wrist, and c) knee. Detection of finger bending at different bending frequencies in d) ambient air and e) underwater. f) Relative resistance changes of MXN_5_/B‐CP under 500 s of continuous bending. g) Relative resistance changes under increasing compressive pressure. h) The patient who is unable to talk can send signals through body language, such as i) SOS signals.

In addition to their electrical and mechanical robustness, the suitability of MXene papers for wearable applications hinges on their environmental adaptability. To this end, the waterproof and hydrophobic properties of the porous Ecoflex‐coated MXN_5_/B‐CP were first investigated to determine its performance under moisture‐sensitive and real‐world operating conditions. The surface wettability of MXN_5_/B‐CP has been tested with five common liquids in our daily life, i.e., water, ethanol, milk, green tea, and coffee, as demonstrated in **Figure** **4**a. While the contact angle for ethanol was a mere 31°, indicating a strong affinity for alcohol‐based solvents, water and other water‐based liquids exhibited significantly higher contact angles (101° for water, 81° for milk, 90° for green tea, and 85° for coffee), highlighting the limited wettability of the coated paper toward water‐based liquids.^[^ ^57^ ^]^ Consequently, the Ecoflex‐coated MXN_5_/B‐CP paper demonstrates moderate hydrophobicity, which helps to prevent water penetration (further discussed in **Figure** **5**e). An evaporation test was conducted to analyze the moisture permeability of the waterproofed MXN_5_/B‐CP (Figure 4b). Equal amounts of water were sealed inside bottles using waterproofed MXN_5_/B‐CP and Ecoflex, and left unsealed as a control. The bottles were left at room temperature for 8 days, and their weight loss was measured to evaluate moisture transmission. There is a significant difference in evaporation rates between an open bottle and porous Ecoflex and waterproofed MXN_5_/B‐CP. Both the porous Ecoflex and the porous Ecoflex‐coated MXN_5_/B‐CP exhibit a similar amount of moisture penetration, with average breathability of 167.97 g m^−2^ day^−1^ (Ecoflex) and 173.90 g m^−2^ day^−1^ (Ecoflex‐coated MXN_5_/B‐CP), indicating a balance between waterproofing and gas permeability necessary for skin breathability.

The joule heating effect of the MXN_5_/B‐CP paper was further analyzed. When subjected to an external voltage, the composite exhibits a controllable and rapid thermal response. As shown in Figure 4c,e, increasing the voltage from 1 to 4V resulted in temperature rises from 30.9 to 34.4, 50.7, and 79.6 °C, with corresponding IR thermal images confirming the uniform heating profile across the sample. Conversely, when the voltage was abruptly cut, a rapid decline in temperature confirmed the reversible thermal response. The stability testing of the MXN_5_/B‐CP was evaluated by applying a 3V voltage for 30 cycles (Figure 4d). Notably, the results demonstrated consistent and repeatable heating behavior throughout all cycles, thus reinforcing the reliability and stability of the MXN_5_/B‐CP. This controllable and efficient Joule heating behavior makes the MXN_5_/B‐CP electrode suitable for thermotherapy applications.

An additional advantage of MXN_5_/B‐CP sensors lies in their excellent customizability. Unlike traditional flexible devices constructed on polymer substrates, the MXN_x_/B‐CP can be easily cut and trimmed into various sizes and shapes using scissors, enabling personalization to meet individual requirements. By utilizing a laser cutting machine, the kirigami technique is employed to create ribbon‐like patterns (Figure 4f), thereby enhancing the flexibility and stretchability of the MXN_5_/B‐CP paper. Notably, the kirigami‐pattern MXN_5_/B‐CP could be stretched up to 30 times its original length without structural integrity or functionality, making it ideal for high‐mobility wearable applications (Figure 4g).^[^ ^58^ ^]^ Furthermore, our MXN_5_/B‐CP also exhibits excellent fire resistance. In contrast to pure CNF paper, the MXN_5_/B‐CP is resistant to burning (Figure 4h) and can withstand direct exposure to a commercial lighter flame for up to half a minute without catching fire.

The biocompatibility of the MXN_5_/B‐CP was evaluated through a cell viability assay, as illustrated in Figure 4i. The assay employs a dual‐staining technique where cells are identified based on cell nucleus staining (Hoechst 33342), and dead cells are marked by red fluorescence (PI). Quantitative analysis reveals a high cell viability rate of 98.9 ± 0.3%, suggesting that the MXN_5_/B‐CP displays no cytotoxicity. This finding underscores the nontoxic nature of the MXN_5_/B‐CP composite material, which is crucial for its potential use in wearable and implantable biomedical devices. The absence of significant cell death supports the biocompatibility of the sensor, rendering it suitable for real‐time human motion monitoring applications. From a sustainability standpoint, the MXN_5_/B‐CP sensor demonstrated effective degradability in oxidative environments. By immersing MXN_5_/B‐CP (without waterproof coating) in 1 wt.% H_2_O_2_ solution (Figure 4j) and phosphate buffer solution (PBS) (Figure S4, Supporting Information), the MXN_5_/B‐CP gradually decomposed within 240 min and 72 h, ultimately resulting in complete structural disintegration and permanent elimination of the paper.

### 2.4 Sensing Performance and Application in Wearable Devices

The exceptional flexibility, sensitivity, and stability of the conductive MXN_5_/B‐CP sensor position it as an ideal candidate for wearable electronics in human body movement monitoring. Real‐time strain sensing was demonstrated through five repeated measurements on a volunteer's wrist, elbow, and knee (Figure 5a–c). During similar joint‐bending motions, the sensor consistently detected distinct ΔR/R_0_ peaks corresponding to each bending movement. The baseline ΔR/R_0_ values for different joint movements are summarized in Figure S5a (Supporting Information), where the average values with error bars for both baselines and peak responses are presented. The baseline values remained stable, with only limited inconsistency observed. Variations in peak values are likely due to natural differences in the volunteer's motion, such as slight changes in bending angle, speed, or pressure. Despite these joint‐specific differences, the MXN_5_/B‐CP sensor exhibited stable, repeatable, and reliable responses, accurately capturing motion patterns during repeated bending under consistent testing conditions. The sensor also accurately identifies and differentiates between varying finger bending frequencies ranging from 0.5 to 2 Hz (Figure 5d), demonstrating an exceptional capability to recognize diverse human motions.

To assess the sensor's waterproof performance, we repeated the frequency‐dependent bending experiments under submerged conditions, where the MXN_5_/B‐CP was adhered to a finger joint (Figure 5e) and subjected to repeated flexion and extension at different bending frequencies. The relative resistance changes under water closely matched those recorded in the air condition, with only minor fluctuations attributed to slight variations in finger extension during each cycle. To further validate the underwater stability of the MXN_5_/B‐CP sensor, its conductivity was confirmed by successfully lighting an LED during underwater bending (Video S1, Supporting Information). Then we evaluated its finger bending sensing performance before (Figure S5b, Supporting Information) and after (Figure S5c, Supporting Information) soaking in water for 0.5 h. As the figures demonstrate, the finger‐bending sensing performance remained similar, with only minor signal variations observed (Figure S5d, Supporting Information), confirming its underwater stability.

Despite these small differences, the sensor continued to function reliably, demonstrating its potential for applications in moisture‐rich or underwater environments, an attribute crucial for reliable operation in practical wearable devices. This should be ascribed to the structural design of the paper sensor, where the functional MXene is effectively encapsulated by the CNF matrix and the Ecoflex layer, providing robust protection against water ingress.

In terms of long‐term performance, Figure 5f shows that the sensor demonstrates a stable sensing pattern over 500 s of continuous bending, maintaining consistent sensitivity and recoverability. This retention performance underscores the sensor's suitability for a broad array of practical applications, accommodating different dynamic movements anticipated in real‐world settings. The pressure‐sensing performance in Figure 5g reveals a 60% increase in resistance as the applied compressive pressure rises from 0 to 10 kPa, with saturation observed beyond 10 kPa up to 50 kPa, suggesting that the MXN_5_/B‐CP sensor can maintain its structural integrity and electrical functions even under considerable force. Beyond motion and pressure detection, the MXN_5_/B‐CP sensor has been successfully implemented as an alternative communication system for those unable to speak. As depicted in Figure 5h, specific motion patterns, such as Morse‐code SOS (Figure 5i), NTU, and TEL LAB (Figure S6b, Supporting Information), can be transmitted via body movement in emergency situations. The complete Morse code for English letters is shown in Figure S6a (Supporting Information), further demonstrating the potential of this sensor in assistive technologies for nonverbal communication.

### 2.5 Evaluation of EMG Control and Human‐Machine Interaction Capabilities

**Figure 6:**
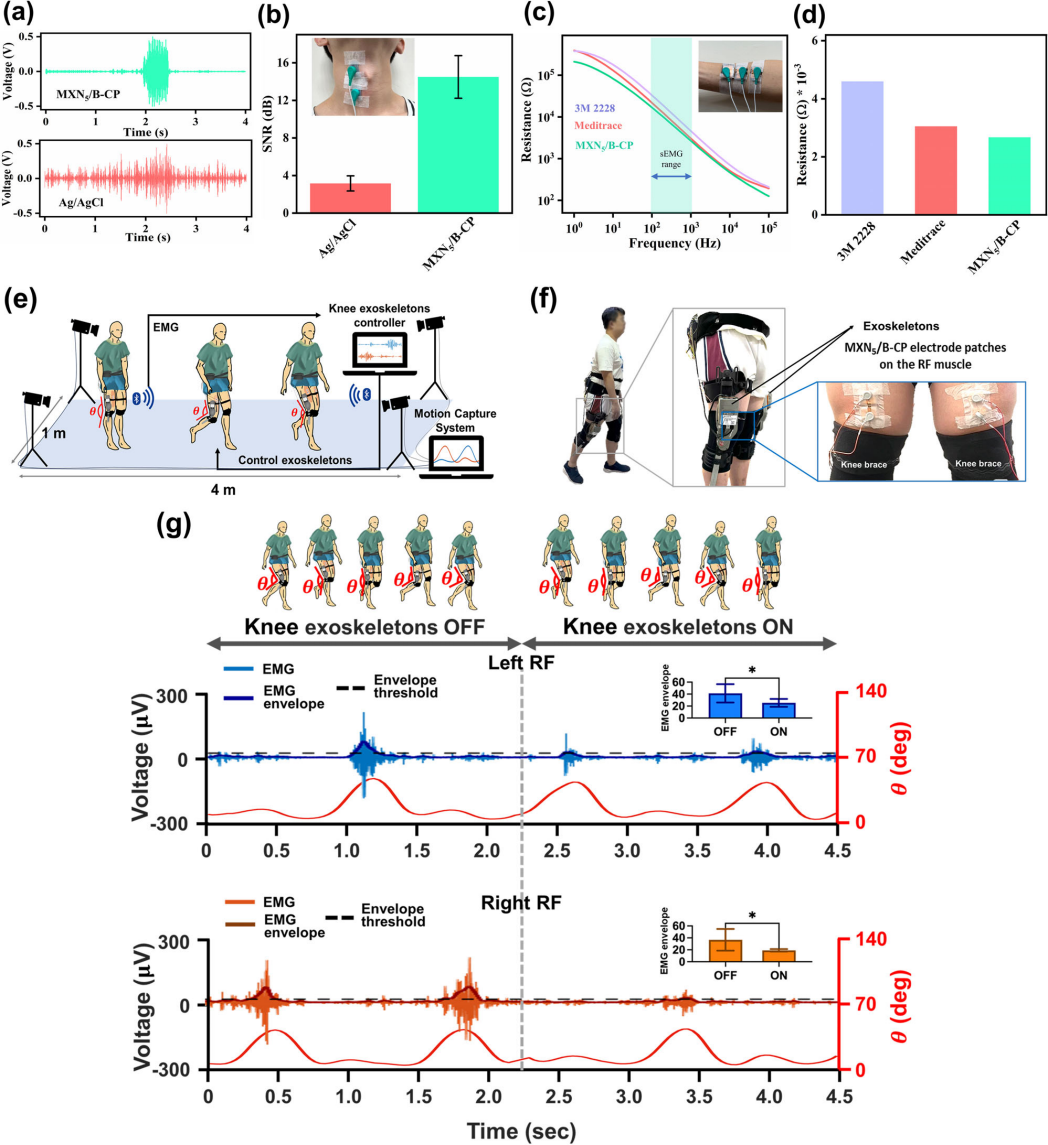
Experimental demonstration of the EMG‐controlled knee exoskeleton system. a) Swallowing EMG signal detected by MXN_5_/B‐CP and commercial Ag/AgCl electrodes. b) SNR comparison of the two electrodes during swallowing. The inset shows the digital photo of the sensor placement on the throat. c) Frequency‐dependent impedance spectra for 3M 2228, Meditrace, and MXN_5_/B‐CP in the 1–10^5^ Hz range. The shaded region highlights the typical EMG frequency range. d) Impedance values of the electrodes in the EMG frequency of 1 kHz. e) Schematic of the experimental setup showing a 4‐meter walking path, with motion capture using a four‐camera system and EMG signal transmission via Bluetooth to the controller. EMG signals from the RF muscles were utilized to activate the exoskeleton. Motion data were captured synchronously at 30 Hz for kinematic analysis. f) Close‐up of the knee exoskeleton system, including the MXN_5_/B‐CP based electrode patches secured to the RF muscles using knee braces to minimize motion artifacts. The power supply and Bluetooth‐based EMG transmission module were positioned on the lower back for mobility. g) EMG and kinematic analysis during gait. Left and right RF muscle activity (voltage signals and their envelopes) and knee joint angles (θ) are compared between conditions with the exoskeleton OFF and ON. The threshold for gait onset detection was marked by three times the baseline envelope standard deviation. Statistical analysis revealed a significant reduction in RF muscle activation (^*^ *p* < 0.05) during exoskeleton‐assisted walking, while joint angle patterns remained stable, demonstrating consistent biomechanics.

To verify its commercial potential, we fabricated two EMG electrode sensors based on MXN_5_/B‐CP and commercial Ag/AgCl, which have been affixed to a volunteer's throat to monitor swallowing motions. As illustrated in Figure S7 (Supporting Information), the device is composed of a button, an Ecoflex layer, carbon tape, and the MXN_5_/B‐CP electrode. The EMG signals in **Figure** **6**a indicate that the MXN_5_/B‐CP electrode exhibits a lower baseline noise during water swallowing compared to commercial Ag/AgCl electrodes. Remarkably, the MXN_5_/B‐CP sensor achieves a more than fourfold improvement in signal‐to‐noise ratio (SNR) compared to commercial Ag/AgCl, coupled with much lower noise in the time domain (Figure 6b). Figure 6c presents an impedance (Z) versus frequency plot comparing three commercial electrode materials: 3M 2228 tape, Meditrace electrodes, and MXN_5_/B‐CP, ranging from 10 to 100 kHz. Within the typical frequency range for EMG signals (highlighted in the shaded area), our MXN_5_/B‐CP sensor demonstrates significantly lower impedance at the 1 kHz frequency spectrum (Figure 6d), suggesting enhanced electrical coupling and reduced signal interference. These findings highlight the MXN_5_/B‐CP potential as a more efficient and reliable alternative to biomedical applications, particularly in tracking and analyzing complex human movements for bio‐signal acquisition applications.

To further evaluate the real‐time human‐machine interaction capabilities, we implemented the MXN_5_/B‐CP electrodes in the control of a knee exoskeleton (KneeBO, FREE Bionics Taiwan Inc.). The system aimed to enhance gait stability and reduce neuromuscular effort during walking. Control signals were derived from EMG recordings of the rectus femoris (RF) muscles using our electrodes. As shown in Figure 6e, three healthy male participants (aged 23–28 years, BMI 17.8–24.1) walked along a 4‐meter path under two conditions: exoskeleton deactivated (OFF) and activated (ON). All experimental procedures were conducted under the approval of the Taipei Veterans General Hospital Institutional Review Board (IRB‐TPEVGH No. 2024‐08‐003AC), and written informed consent was obtained from all participants. EMG and kinematic data were collected using a synchronized setup (EMG: 1000 Hz via Bluetooth; motion capture: 30 Hz). Electrodes were secured with braces to minimize artifacts (Figure 6f), and bilateral exoskeletons were worn with wireless EMG modules attached at the lower back. EMG data underwent a real‐time processing pipeline (detrending, notch filtering, 4th‐order Butterworth bandpass: 40–400 Hz, envelope extraction via 9 Hz low‐pass filter). Gait onset was triggered by exceeding a threshold of 3 × standard deviation of baseline activity during a 2‐s standing period (Figure 6g). Knee joint angles were analyzed offline using OpenSim software to reconstruct 3D skeletal data.^[^ ^59^, ^60^, ^61^ ^]^

To ensure a high‐quality EMG signal during this pilot phase, participants were selected within a lean‐to‐medium BMI range (17.8–24.1), as subcutaneous fat thickness is known to attenuate EMG amplitude and shift spectral content.^[^ ^62^, ^63^, ^64^ ^]^ This range helped minimize variability due to adipose‐related signal loss,^[^ ^64^, ^65^ ^]^ thereby improving the reliability of signal comparison with commercial electrodes. We quantified system latency to assess real‐time viability. Total latency from signal acquisition to motor actuation was ≈100 ± 8 ms. This includes wireless transmission (39 ± 5 ms), PC‐side processing (19 ± 3 ms), command transmission to the controller (11 ± 2 ms), and motor response time (≈30 ms). These values fall within the widely accepted 100–150 ms threshold for EMG‐driven systems,^[^ ^66^, ^67^ ^]^ ensuring responsive control during locomotion. In this study, we employed two independent, commercially available knee exoskeleton modules, one for each leg. Each module was controlled via motor commands transmitted from a remote PC, which performed real‐time processing of EMG signals recorded from the user's RF muscles to generate control inputs. Importantly, no software‐based synchronization or mechanical coupling was implemented between the two exoskeletons to enforce interlimb coordination. Instead, the system leveraged the user's intrinsic biological mechanisms, specifically, central pattern generators (CPGs), which are spinal neural circuits responsible for producing rhythmic locomotor activity.^[^ ^68^ ^]^ By relying on these endogenous neuromuscular rhythms,^[^ ^69^ ^]^ the system enabled natural interlimb coordination without external constraints.^[^ ^70^ ^]^ Therefore, this biologically congruent control strategy reduced the likelihood of gait desynchronization and supported more natural and stable locomotion patterns.^[^ ^71^ ^]^ To safeguard users during operation, a multitiered safety framework was implemented. Hardware protection included FAULHABER motors (BP4, Faulhaber AG, Switzerland) in the knee exoskeleton with built‐in detection of overcurrent, thermal overload, and short circuits. Simultaneously, the control software enforced joint‐level torque and angle constraints (+5 N·m to –10 N·m; –105° to 180°), preventing excessive biomechanical loading. Together with the system's low‐latency EMG feedback and CPG‐based coordination, this design enabled safe and intuitive interaction. Notably, statistical analysis using the Mann‐Whitney test (p < 0.05) revealed a significant reduction in RF activation during exoskeleton‐assisted walking. This finding confirms decreased neuromuscular demand, aligning with the primary goal of our exoskeleton support systems to reduce metabolic cost and muscle fatigue while preserving cyclic knee kinematics and biomechanical fidelity. Meanwhile, knee joint angles exhibited stable and cyclic patterns across conditions, underscoring our exoskeleton system's ability to maintain consistent biomechanics.

The preservation of natural, repeatable knee kinematics indicates that the assistance provided by the exoskeleton was well‐coordinated with the users’ intrinsic motor patterns, avoiding any disruption to normal gait dynamics. Such biomechanical fidelity is crucial for ensuring user safety and preventing compensatory movements that could lead to joint overloading or long‐term musculoskeletal issues. Video S2 (Supporting Information) demonstrates the real‐time performance of the MXN_5_/B‐CP based wearable sensor for detecting muscle activity. The video highlights the sensor's stable signal output, fast response, and potential for integration into real‐time human‐machine interface systems. These findings validate the functionality of MXN_5_/B‐CP‐fabricated EMG electrodes in accurately capturing muscle activity, facilitating real‐time exoskeleton control, and promoting biomechanically stable gait patterns during assisted walking. Moreover, the successful wireless transmission, stable electrode performance under dynamic conditions, and effective detection of gait events demonstrate the practical reliability and robustness of MXN_5_/B‐CP electrodes for integration into future smart wearable human‐machine interfaces.

## 3 Conclusion

In conclusion, we introduce a 2D MXene‐integrated bamboo‐derived CNF paper for multifunctional and sustainable wearable electronics. Structural characterizations confirm the successful integration of MXene within the CNF matrix, yielding a tightly bound and uniformly distributed composite. Our MXene paper electrode demonstrates a suite of advantageous properties, including biodegradability, conductivity, gas permeability, waterproofing, and fireproofing properties, underscoring its potential for next‐generation wearable electronics. The MXN_5_/B‐CP exhibited a nonlinear mechanical and piezoresistive response, with Young's modulus increasing from 0.064 MPa at low stress to 1.768 MPa at higher stress levels. Correspondingly, the GF improved from 3.73 in the low strain region to 11.42 in the high strain regime, highlighting the material's sensitivity tuning across different deformation scales. The MXN_5_/B‐CP system effectively measures movements of body parts such as fingers, wrists, elbows, and knees, exhibiting high sensitivity and stable resistance signal detection during continuous bending. Its strain‐sensing properties enable efficient detection of human motions in both air and underwater environments. Additionally, a kirigami pattern enhances the material's stretchability and elongation, while its low cytotoxicity and degradability support safe use and eco‐friendly disposal. Notably, the sensor enables nonverbal communication via signal‐based encoding (Morse code) and demonstrates practical utility in real‐time human‐machine interfaces (exoskeleton control via EMG signals). Overall, this work establishes the MXN_5_/B‐CP sensor as a promising candidate for future wearable electronics, offering a scalable pathway to daily interactions with technology and enhancing specialized environments. In future work, we plan to explore alternative sensing mechanisms of MXN_x_/B‐CP beyond the current approach. In particular, capacitive sensing will be investigated to evaluate its potential for enhanced sensitivity, reduced power consumption, and suitability for different types of stimuli. This exploration may open up new application avenues and further improve the multifunctionality of MXN_x_/B‐CP‐based wearable devices.

## Conflict of Interest

The authors declare no conflict of interest.

## Supporting information



Supporting Information

Supplemental Video 1

Supplemental Video 2

## Data Availability

The data that support the findings of this study are available from the corresponding author upon reasonable request.
