# Supplementary material for: Biodegradable MXene‐Bamboo Cellulose Paper Electrodes for Green Wearable Sensing and Exoskeleton Control
Source: Adv Sci (Weinh). 2025 Sep 11;12(45):e09554. doi: 10.1002/advs.202509554 (PMC12677603; doi:10.1002/advs.202509554)
Supplement: Supplementary file 1 — Supporting Information [file ADVS-12-e09554-s002.docx]

Supporting Information

**Biodegradable MXene-bamboo cellulose paper electrodes for green wearable sensing and exoskeleton control**

*Tung-Li Hung, Chien-Yu Huang, Chun-Ho Lin, Yu-Chen Wei, Yung-Jung Hsu, Jr-Hau He, Yun-Ting Kuo, An-Yu Huang, You-Yin Chen, You-Rong Lin, Clemens M. Franz, Chia-Hao Kuo, Pulikkutty Subramaniyan, Shan-Chu Yu, Xinwei Guan*, Tzu-En Lin**

1. **Chemicals**

MXene Ti_3_C_2_T_x_ was purchased from XinXi-technology. Both component A and component B of Ecoflex were purchased from BASF Corporation. The bamboo fiber paper was purchased from Zhonghua Brush Pen Store. Sodium hydroxide (98.5%) was purchased from ACROS. (2,2,6,6-Tetramethylpiperidin-1-yl)oxyl or (2,2,6,6-tetramethylpiperidin-1-yl)oxidanyl (TEMPO) (99%) and sodium hypochlorite (11-15%) were purchased from Thermo Scientific. Sodium chlorite (80%) was purchased from SHOWA. Glacial acetic acid (99.7%) was purchased from ECHO. Mono and disodium hydrogen phosphate (99%) was purchased from SIGMA. All experiments were conducted using deionized water (DI) (18.25 MΩ·cm at 25 °C), supplied by Sartorius.

1. **Synthesis procedure**
   1. *Synthesis of bamboo-derived cellulose nanofiber*

A 500 ml pulp of recycled bamboo fiber paper was prepared at a concentration of 2 wt%, followed by adding 5 wt% sodium hydroxide. The pulp solution was then continuously stirred at 80°C for 2 hours. Afterward, vacuum filtration was performed, and the pulp was washed multiple times with DI water to remove any unreacted sodium hydroxide. Finally, the fibers were dried in an oven at 60°C. In the second stage, 10 g of dried bamboo fiber pulp was dispersed in 160 ml of DI water, followed by the addition of 80% sodium hypochlorite and 200 μl of glacial acetic acid. The mixture was then heated at 80°C for 1 hour. This step aimed to remove lignin from the pulp, improving the purity of the nanocellulose. The third stage involved TEMPO-mediated oxidation. The treated 5 g of fiber pulp was dispersed in a 0.05 M phosphate buffer solution (PBS) (500 ml, pH 7) containing 200 mg of TEMPO [S1, S2]. Then, 4 g of sodium chlorite and 20 ml of 4% sodium hypochlorite solution were added, and the reaction container was immediately sealed to maintain stable reaction conditions. The solution was heated at 60°C for 18 hours. Upon completion, chemical waste was removed by suction filtration, and the fibers were washed and filtered three times with DI water. Finally, the filtered bamboo paper fibers were adjusted to a 2.5 wt% aqueous solution and homogenized three times for 10 minutes each with 5-minute intervals. The homogenized paper fibers were then centrifuged at 4400 rpm for 30 minutes, and the supernatant was collected. Due to the low concentration of the supernatant, it was heated to evaporate excess water until a cellulose slurry was obtained, which was then dried in an air oven at 60°C overnight.

- 1. *Synthesis of single-layer MXene nanosheets*

Multilayered Ti_3_C_2_T_x_ MXene was exfoliated into single-layer nanosheets via ultrasonic treatment. In the experiment, varying amounts of MXene (0 mg, 0.1 mg, 1 mg, 2 mg, 5 mg, 8 mg) were dispersed in 3 ml of DI water. Each dispersion was subjected to ultrasonic treatment using a probe-type ultrasonicator at 100 W for 1 hour under an ice bath to achieve effective exfoliation.

- 1. *Synthesis of MXNx/B-CP*

3.3 mg of CNF was accurately weighed and added to 3 ml of deionized water, followed by ultrasonication at 100 W for 1 hour to ensure uniform cellulose fiber dispersion. The dispersed fibers enhance the adhesion of MXene in subsequent steps and ensure the uniformity of the composite material. After sonication, the pre-prepared MXene aqueous solution was added, and the mixture was sonicated again for 1 hour in an ice bath. The MXene/CNF mixture was then vacuum-filtered through a 0.22 μm MCE (Mixed cellulose ester) membrane, air-dried, and peeled off from the filter membrane to yield a co-dispersed MXN_x_/B-CP.

- 1. *Fabrication of the waterproof layer of the MXNx/B-CP*

To impart waterproofing while maintaining breathability and skin compatibility, the MXN_x_/B-CP was encapsulated with a porous Ecoflex thin film, a highly elastic, biocompatible silicone material suitable for skin-interfacing electronic devices. In detail, 1 g of fine brown sugar (sieved through an 80-mesh screen) was mixed with 1 ml each of Ecoflex Part A and Part B, and this mixture was then poured into a glass plate and dried in a preheated oven at 50°C for 1 hour to cure the Ecoflex film. Next, the cured film was immersed in water and heated with continuous stirring at 100°C for 12 hours to remove all sugar particles from the film, thus generating a microporous structure that effectively improves the breathability of the encapsulated sensor. The MXN_x_/B-CP was then precisely cut into rectangular pieces with dimensions of 15 mm × 10 mm. Carbon tape was used to securely connect conductive sewing threads to both ends of the MXN_x_/B-CP. Finally, the sensor was placed between two thin films, and the surrounding area was securely bonded with a mixture of Ecoflex to ensure a tight seal.

- 1. *Fabrication of the surface electromyography (EMG) sensor*

The MXN_x_/B-CP is initially cut to size with precision to meet the button electrode’s requirements. We use carbon tape to secure conductive sewing thread onto one side of the MXN_x_/B-CP. The sewing thread should be attached perpendicular to the MXN_x_/B-CP, ensuring that part of the thread is exposed for easy subsequent connection. Next, 1 ml of Ecoflex Part A and 1 ml of Part B are mixed together and evenly coated over the carbon tape, including the sewing thread. The Ecoflex-coated MXN_x_/B-CP electrode is then placed in a preheated oven at 50°C and baked for 1 hour to complete the curing process. After curing, excess sewing thread is secured by looping it tightly around the button electrode. Finally, the edges are trimmed to achieve a neat appearance and appropriate size.

1. **Characterization**

The morphology and microstructure of the MXN_x_/B-CP samples were observed using a scanning electron microscope (SEM, SU-8010) operated at an accelerating voltage of 15 kV, and corresponding elemental composition and distribution were studied through energy-dispersive X-ray spectroscopy (EDS) mapping. To characterize the surface topography of the fiber structure, atomic force microscopy (AFM) was performed using a Bruker Dimension ICON SPM with a cantilever, and AFM data were processed using Gwyddion software. Crystal structures were investigated by X-ray diffraction (XRD, Empyrean) with a 2θ scanning range from 5 to 70 degrees. The chemical state and functional bonding of the MXN_x_/B-CP were analyzed using X-ray photoelectron spectroscopy (XPS-Thermo Fisher Scientific ESCALAB Xi+ with Al Kα radiation).

Electrical and electromechanical characteristics of MXN/B-CP were carried out using a digital source meter (Keithley 6514), with ΔR/R adopted as the data collection metric. The electrochemical stability was evaluated via linear sweep voltammetry (LSV) using a CHI electrochemical workstation, scanning from –0.5 V to 0.5 V at a scan rate of 0.02 V/s. A power supply (GPD-4303S) was used to apply voltages ranging from 1 V to 4 V to the MXN_x_/B-CP. An infrared thermometer (E5-XT) was employed to observe the temperature changes, allowing for the evaluation of the Joule heating effect. The MXN_x_/B-CP was immersed in 1 wt% H_2_O_2_ and PBS solutions to simulate the oxidation degradation process. For the fire resistance test, pure CNF and MXene papers were separately placed over a flame source to observe their burning behavior. The waterproof performance of the Ecoflex film was evaluated by measuring the contact angle of water, milk, green tea, latte, and alcohol on its surface. To analyze the breathability, we used waterproof sensors and MXN_x_/B-CP to seal the openings of sample bottles using quick-drying glue. A control group with an unsealed bottle was also prepared for comparison. All bottles were placed in an oven at 80°C for 5 hours, and the weight change rate of the water in the sample bottles was recorded to evaluate water vapor transmission.

The EMG electrodes were precisely attached to the subject’s throat and secured using breathable tape. The EMG signal collection system used in the experiment consisted of an STM32 NUCLEO-L031K6 microcontroller and a MyoWare muscle sensor. An electrochemical impedance analyzer (EIWP100/EIWP110) was used to measure the impedance performance of 3M 2228, Meditrace, and MXN/B-CP EMG electrodes within an AC frequency range of 1 to 10^5^ Hz. The MXN/B-CP EMG electrodes were used and connected to an exoskeleton device via Bluetooth. The MXN/B-CP EMG electrodes were attached to the subject’s thigh and connected to an EMG signal receiver. The receiver collected the EMG signals and transmitted them to a computer for data processing. The processed signals were then sent back to the exoskeleton device to enable its driving function.

A laser engraver (Beamo) was used to cut the MXN_x_/B-CP into customized Origami patterns. The biocompatibility of MXN_x_/B-CP was identified by propidium iodide staining with dead cells and nucleus staining with Hoechst 33342 (DOJINDO, Japan). U2OS cells (kindly provided by Tsukasa Matsunaga, Kanazawa University, Japan) were cultured in Dulbecco’s Modified Eagle’s Medium (DMEM) supplemented with 10% fetal bovine serum (FBS) and penicillin (10,000 U/ml, Gibco). For cell maintenance, U2OS cells were passaged twice a week and incubated in a humidified incubator at 37°C with 5% CO_2_. A concentration of 5 × 10^4^ cells/ml was seeded in a 96-well plate for 1 day. Subsequently, a 2 mm square piece of MXN/B-C was added into the 96 well plate and co-cultured for 1 day. Quantification of cell viability was performed using a 20× objective fluorescence microscope (BZ-X810 Keyence All-in-one Fluorescence Microscope), and cell counting was carried out with ImageJ using the threshold and particle analysis functions.

1. **Supporting figures and tables**


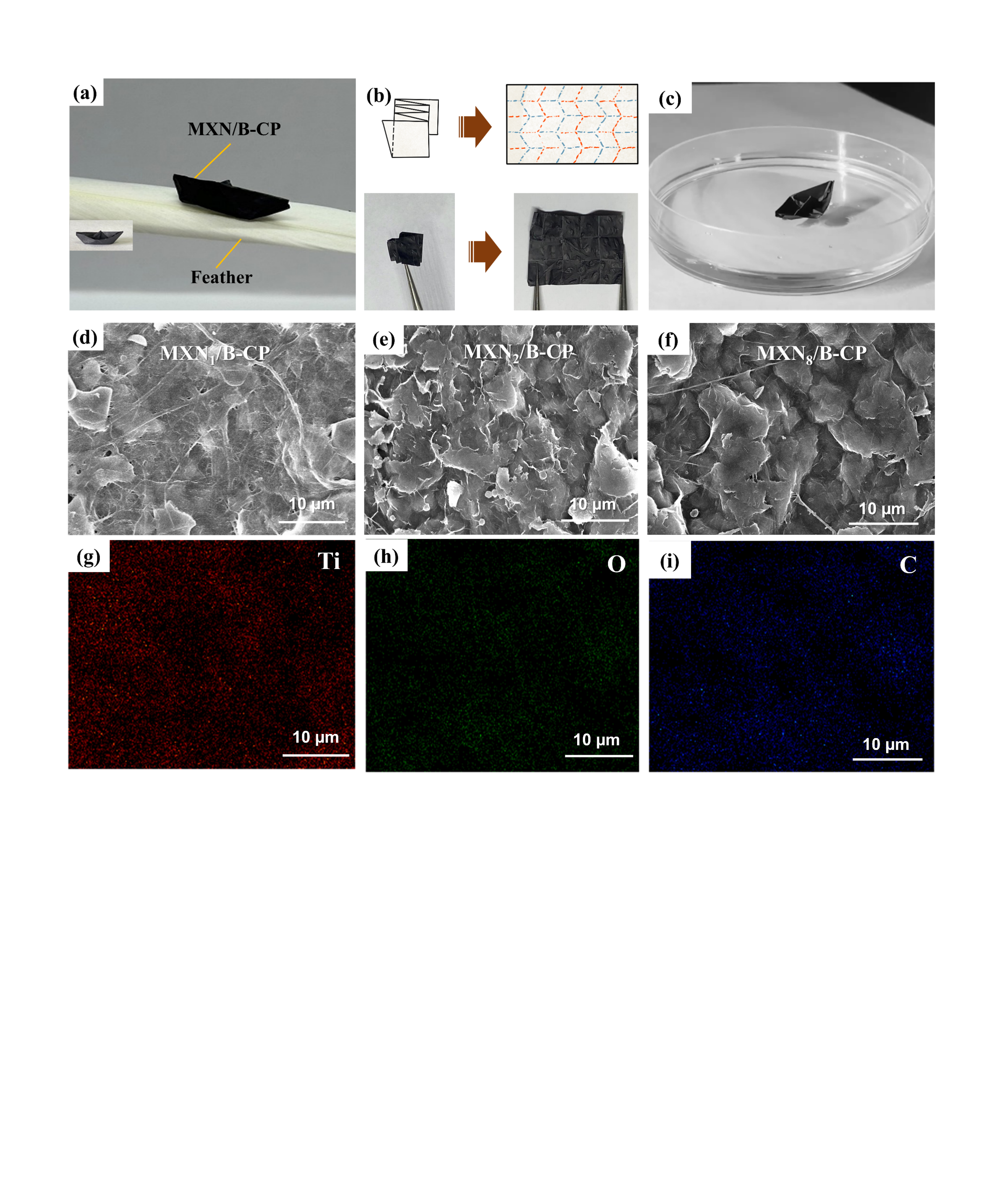


**Figure S1.** (a) An MXN_5_/B-CP boat on a feather. (b) MXN_5_/B-CP folded into Miura structure. (c) MXN_5_/B-CP boat floating on the water. (d-f) SEM micrographs of MXN_x_/B-CP containing different MXene concentrations. (g-i) EDS mapping images of MXN_5_/B-CP (titanium (Ti), oxygen (O), and carbon (C)).


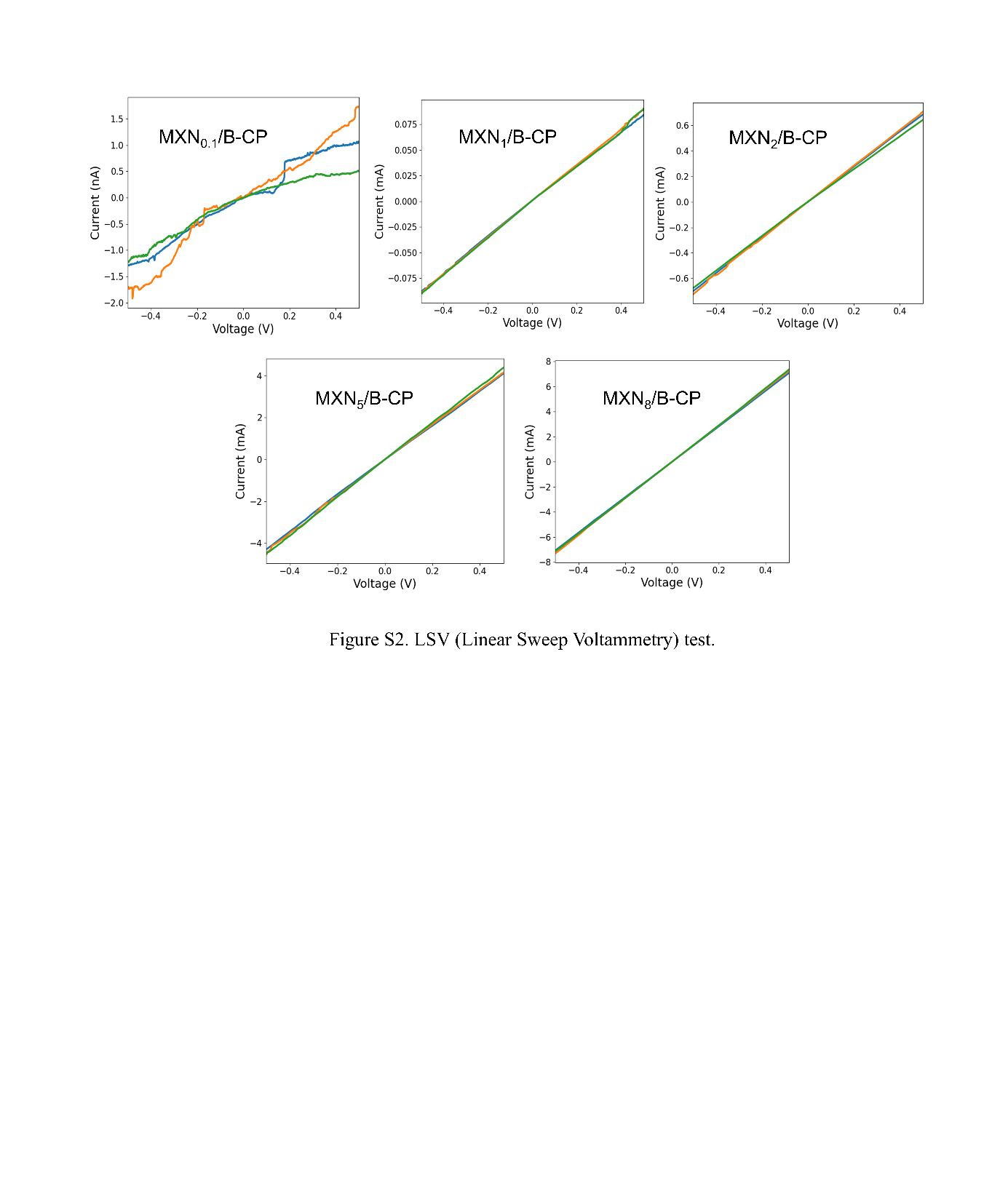


**Figure S2.** LSV (Linear Sweep Voltammetry) analysis of MXN_x_/B-CP with increasing concentration of MXene.


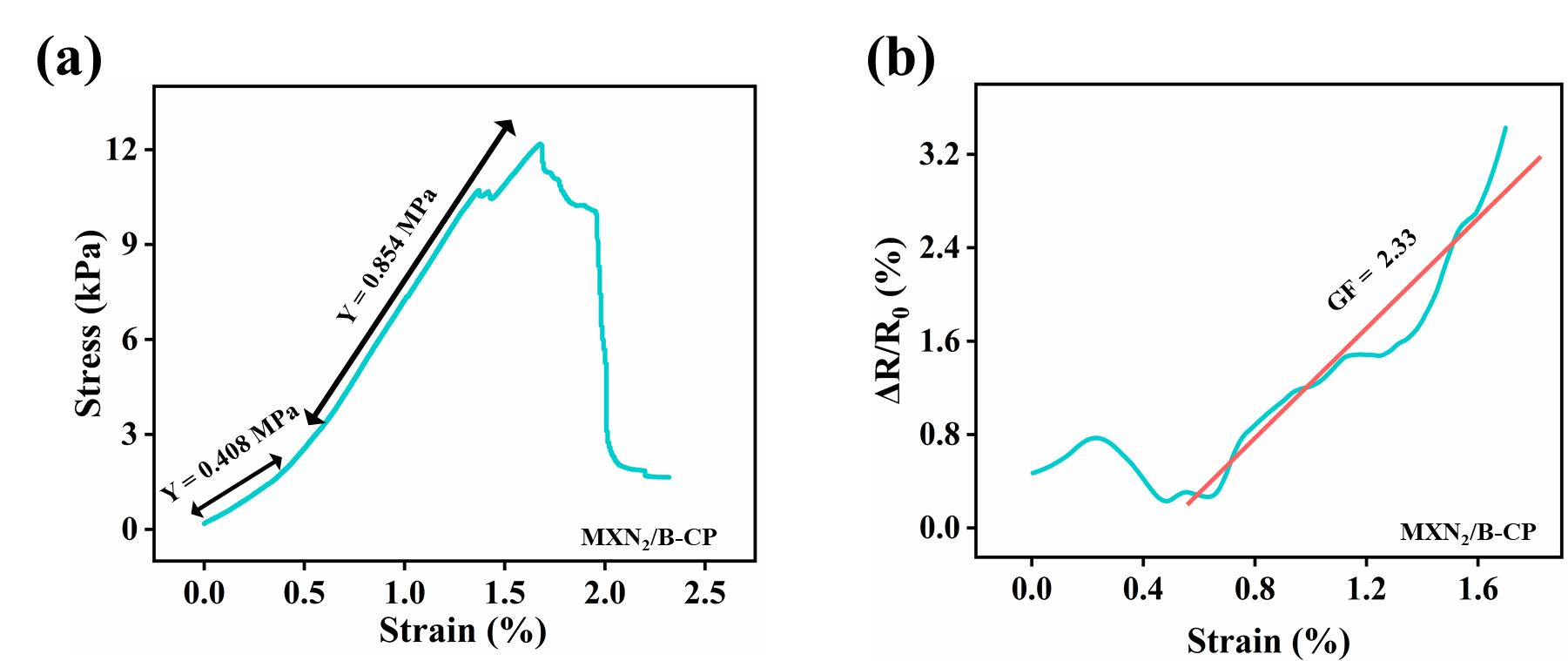


**Figure S3.** a) Stress-strain curve of MXN_2_/B-CP paper. b) Relative resistance changes of MXN_2_/B-CP paper under applied strain.

**Table S1**. Comparison of MXN_5_/B-CP performance metrics with other reported articles.

| **Material** | **Gauge Factor (GF)** | **Strain Range (%)** | **Response Time (ms)** | **Young’s Modulus** | **Durability (cycles)** | **Biodegradability** | **Reference in the paper** |
| --- | --- | --- | --- | --- | --- | --- | --- |
| CNT/PDMS | 1.4 | -1 to +1 | ‑ | ‑ | 35000 | Non-biodegradable | Ref: 46 |
| CB and CMC | 4.3 | 0 to 0.6 | 240 | – | 1000 | Biodegradable | Ref: 47 |
| CNFA_3_@ESF | 6.0 | 0 to10 | 90 | 4.5 GPa | - | Non-biodegradable | Ref: 48 |
| CB/CNTs/TPU | 6.0 | 0 to 3.0 | 248 | – | 2000 | Non-biodegradable | Ref: 49 |
| Hf-SiO_2_/CB/CNT-coated paper | 7.5 | 0 to 0.7 | – | – | 1000 | Biodegradable | Ref: 50 |
| PAV/Cu^2+^-CB-x | 8.24 | 0 to 5 | 410 | 7.41 MPa | 10 | Recyclable | Ref: 51 |
| CNF/X-100-GNP | 9.2 | 0-5 | 61 | 6.18 GPa | 10000 | Non-biodegradable | Ref: 52 |
| MXN_5_/B-CP | 11.42 | 0-2.5 | 120 | 1.768 MPa | 500 | Biodegradable | **This work** |


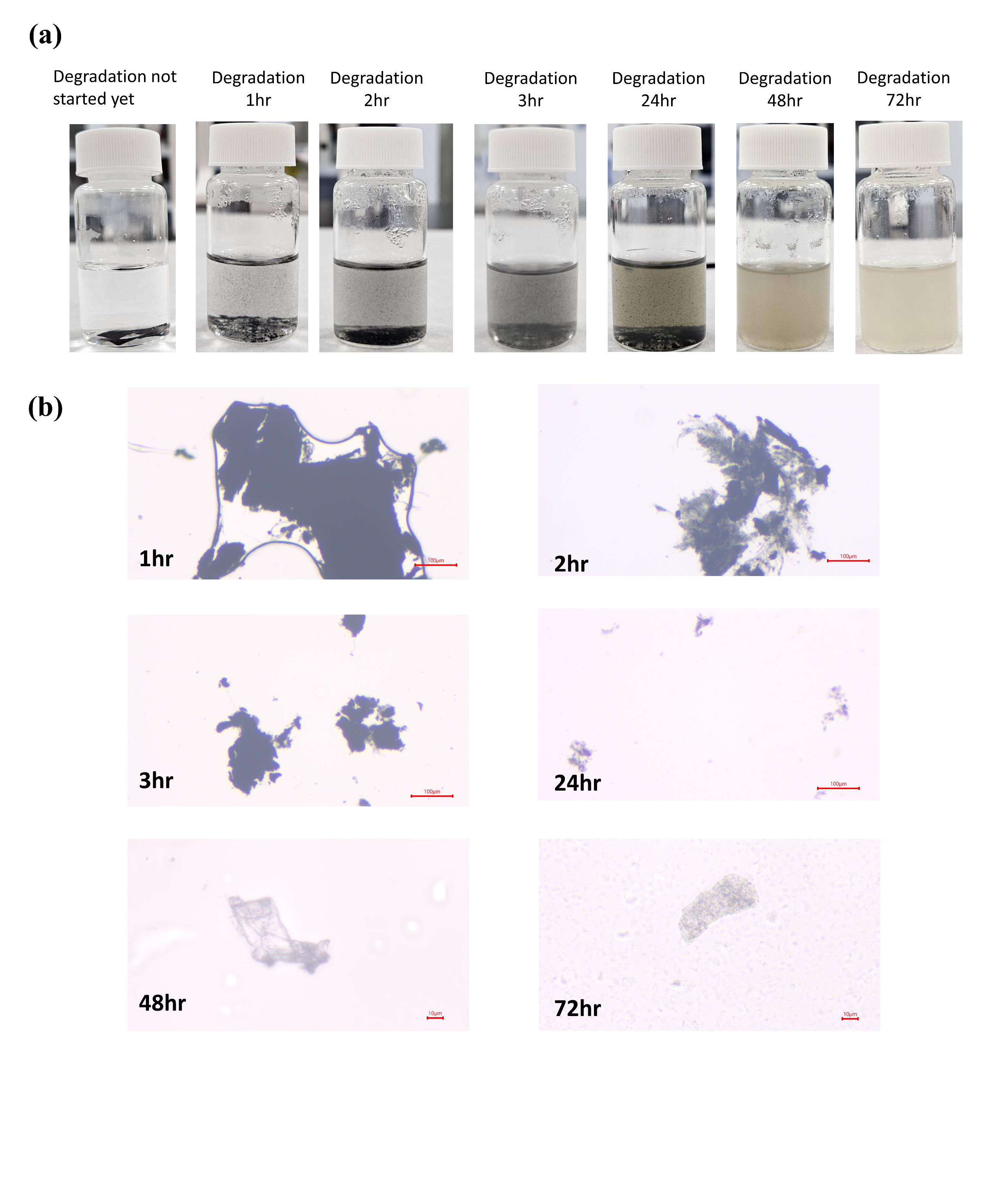


**Figure S4**. (a) Degradation visual photographs and (b) microscopic images of MXN_5_/B-CP maintained under PBS solution at 85 ºC.


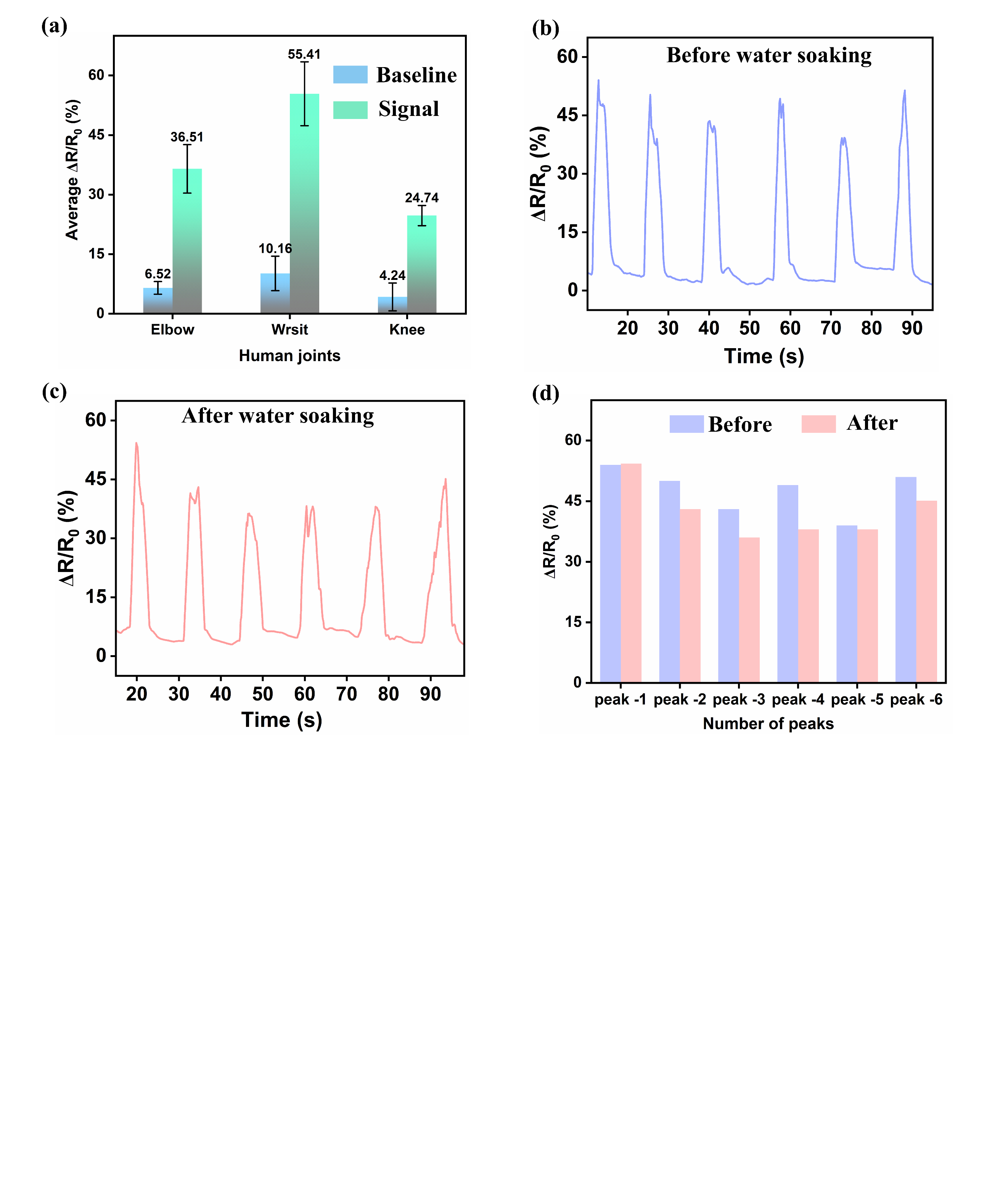


**Figure S5**. (a) Average peak relative resistance changes of bending cycles recorded by the MXN_5_/B-CP sensor in Figure 5a-c for the elbow, wrist, and knee, respectively. (b–c) Relative resistance changes of the MXN_5_/B-CP sensor during finger bending before and after 0.5 h of water soaking. (f) Performance comparison of the MXN_5_/B-CP sensor during finger bending before and after 0.5 h of water soaking, demonstrating stability under wet conditions.

**
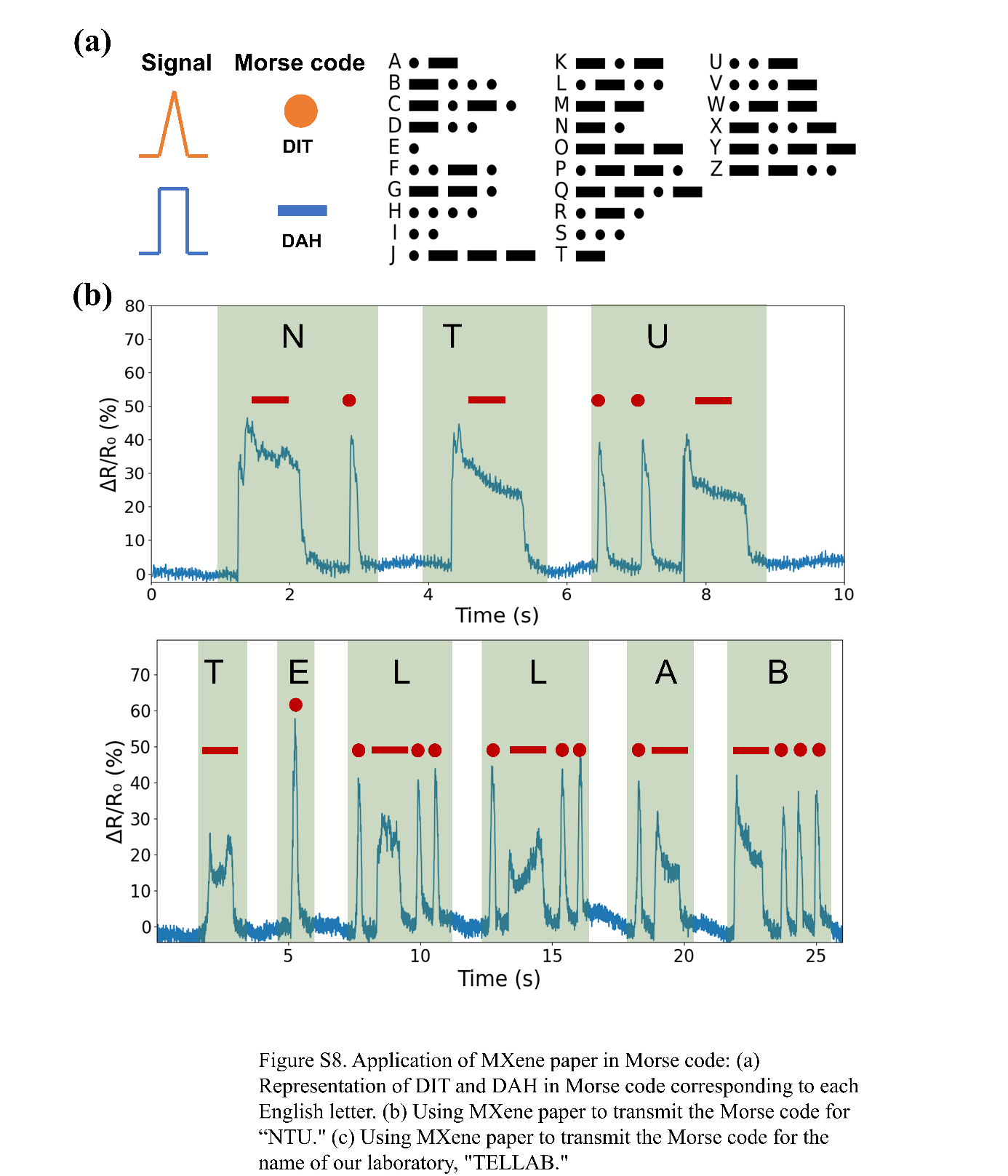
**

**Figure S6**. (a) The signal and Morse code pattern of English letters. (b) The relationship between resistance changes and the Morse code representations of NTU (National Taiwan University) and our group’s name (TEL-LAB).


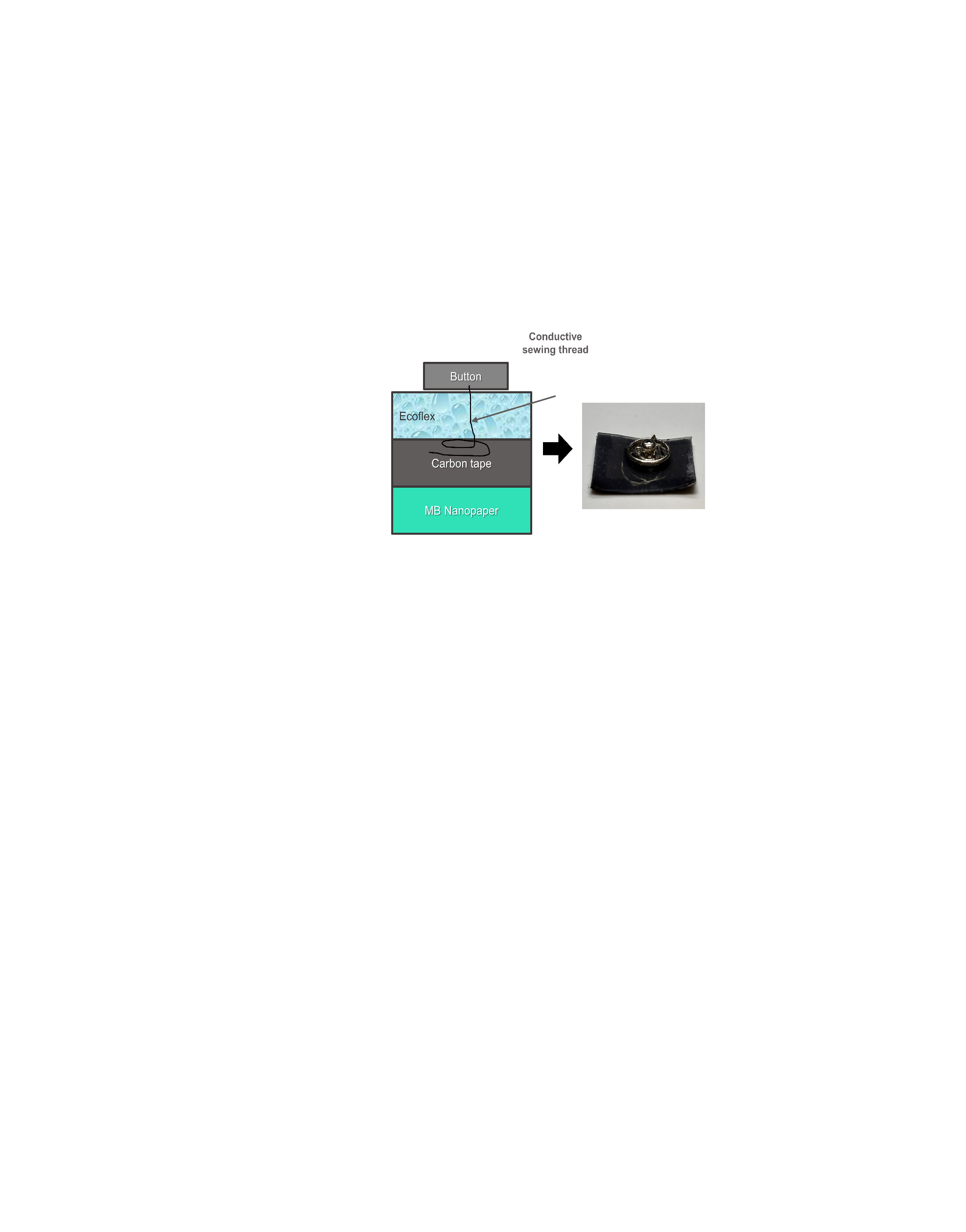


**Figure S7**. (a) Schematic illustration of EMG electrode fragments using MXN_5_/B-CP.

1. **Participants**

This pilot study was conducted in accordance with institutional ethical guidelines and was approved by the Institutional Review Board (IRB) of Taipei Veterans General Hospital (Approval No. 2024-08-003AC). To ensure compliance with ethical standards and to facilitate controlled early-stage validation, the first phase of data collection involved three healthy adult male participants aged 24–26 years. Participants represented a range of body types within the lean to average BMI spectrum (17.8–24.1 kg/m^2^). Prior to enrollment, all individuals were screened to confirm the absence of neuromuscular or orthopedic disorders that might confound surface EMG measurements. Detailed demographic and physiological information is provided in **Table S2**.

**Table S2**. Participant information.

| Participant ID | Age | Gender | BMI Category | BMI Range | Physical Condition | Health Status |
| --- | --- | --- | --- | --- | --- | --- |
| P01 | 24 | Male | Average Body Type | 22.3 | Healthy, physically active | No known neuromuscular or orthopedic disorders |
| P02 | 26 | Male | Average Body Type | 24.1 | Healthy, recreationally active | No known neuromuscular or orthopedic disorders |
| P03 | 25 | Male | Lean Body Type | 17.8 | Healthy, underweight but active | No known neuromuscular or orthopedic disorders |

1. **Supporting Video**

The supporting videos have been attached separately.

**Video S1.** Real-time demonstration of LED illumination during underwater bending using the MXN_5_/B-CP sensor.

**Video S2.** Real-time demonstration of the MXN/B-CP based wearable sensor for detecting upper-arm muscle activity. The user performs muscle contractions while wearing the flexible sensor, and the corresponding signal output is displayed live on the screen, highlighting the sensor’s responsiveness and potential for applications in human-machine interaction.

1. **References**
2. R. Tanaka, T. Saito, A. Isogai, Int. J. Biol. Macromol. 2012, 51, 228.
3. T. Saito, M. Hirota, N. Tamura, S. Kimura, H. Fukuzumi, L. Heux, A. Isogai, Biomacromolecules 2009, 10, 1992.
